# Degradation Potential of Xerophilic and Xerotolerant Fungi Contaminating Historic Canvas Paintings

**DOI:** 10.3390/jof10010076

**Published:** 2024-01-18

**Authors:** Amela Kujović, Cene Gostinčar, Katja Kavkler, Natalija Govedić, Nina Gunde-Cimerman, Polona Zalar

**Affiliations:** 1 Department of Biology, Biotechnical Faculty, University of Ljubljana, Jamnikarjeva 101, SI-1000 Ljubljana, Slovenia; amela.kujovic@bf.uni-lj.si (A.K.); cene.gostincar@bf.uni-lj.si (C.G.); natalija.govedic@gmail.com (N.G.); nina.gunde-cimerman@bf.uni-lj.si (N.G.-C.); 2 Institute for the Protection of Cultural Heritage of Slovenia, Poljanska 40, SI-1000 Ljubljana, Slovenia; katja.kavkler@zvkds.si

**Keywords:** enzymes, biodeterioration, moulds, oil paintings, *Aspergillus*, *Penicillium*, *Cladosporium*

## Abstract

Fungi are important contaminants of historic canvas paintings worldwide. They can grow on both sides of the canvas and decompose various components of the paintings. They excrete pigments and acids that change the visual appearance of the paintings and weaken their structure, leading to flaking and cracking. With the aim of recognizing the most dangerous fungal species to the integrity and stability of paintings, we studied 55 recently isolated and identified strains from historic paintings or depositories, including 46 species from 16 genera. The fungi were categorized as xero/halotolerant or xero/halophilic based on their preference for solutes (glycerol or NaCl) that lower the water activity (a_w_) of the medium. Accordingly, the a_w_ value of all further test media had to be adjusted to allow the growth of xero/halophilic species. The isolates were tested for growth at 15, 24 °C and 37 °C. The biodeterioration potential of the fungi was evaluated by screening their acidification properties, their ability to excrete pigments and their enzymatic activities, which were selected based on the available nutrients in paintings on canvas. A DNase test was performed to determine whether the selected fungi could utilize DNA of dead microbial cells that may be covering surfaces of the painting. The sequestration of Fe, which is made available through the production of siderophores, was also tested. The ability to degrade aromatic and aliphatic substrates was investigated to consider the potential degradation of synthetic restoration materials. Xerotolerant and moderately xerophilic species showed a broader spectrum of enzymatic activities than obligate xerophilic species: urease, β-glucosidase, and esterase predominated, while obligate xerophiles mostly exhibited β-glucosidase, DNase, and urease activity. Xerotolerant and moderately xerophilic species with the highest degradation potential belong to the genus *Penicillium*, while *Aspergillus penicillioides* and *A. salinicola* represent obligately xerophilic species with the most diverse degradation potential in low a_w_ environments.

## 1. Introduction

Artistic canvas paintings often present an important part of the cultural heritage of a country and must therefore be preserved and, if damaged, restored. Biodeterioration is frequently related to inadequate or poorly controlled storage conditions, but it can also occur in modern facilities such as depositories or museum exhibition rooms due to inappropriate heating, ventilation, and air conditioning (HVAC) systems or, in extreme cases, accidental flooding events. Materials introduced during conservation–restoration activities can sometimes promote the process of biodeterioration. The resulting microbial growth and activity can lead to aesthetic changes, mechanical weakening or chemical destruction of the artwork, e.g., staining or discoloration, textural changes, loss of elasticity, deformation, and damage to the textile canvas support [1,2].

Fungal contamination of canvas paintings is common due to the complex and mainly organic, easily degradable materials used as painting components [2,3]. A canvas painting consists of (i) a mechanical support (canvas, wood); (ii) ground layer (animal glue, chalk, a mixture of gypsum or calcium carbonate and glue or flour, oil, and pigments); (iii) paint layers (pigments, drying oil, egg yolk, casein, gums, synthetic resins, such as alkyds, acrylics or ketones) [4,5,6]; and (iv) a cover layer of varnish for protection (dammar, mastic, shellac, or synthetic resins) [4,6,7,8]. Also, conservation–restoration interventions use preferably organic materials [2]. Nevertheless, there is little information on microbial biodeterioration of these materials [2,3,7,9,10,11]. Only a few studies so far have focused on the microbial enzymes responsible for degradation of canvas paintings [11,12,13], and they were based on rapid, low-cost plate assays [14,15]. The fungal genera recognized for their broad range of enzymatic activities were *Aspergillus*, *Cladosporium*, and *Penicillium* [14,15]. In addition, representatives of the these genera can secrete organic acids and pigments [11,14,15,16], which can irreversibly change the appearance and structure of canvas paintings [17,18].

**Figure 1:**
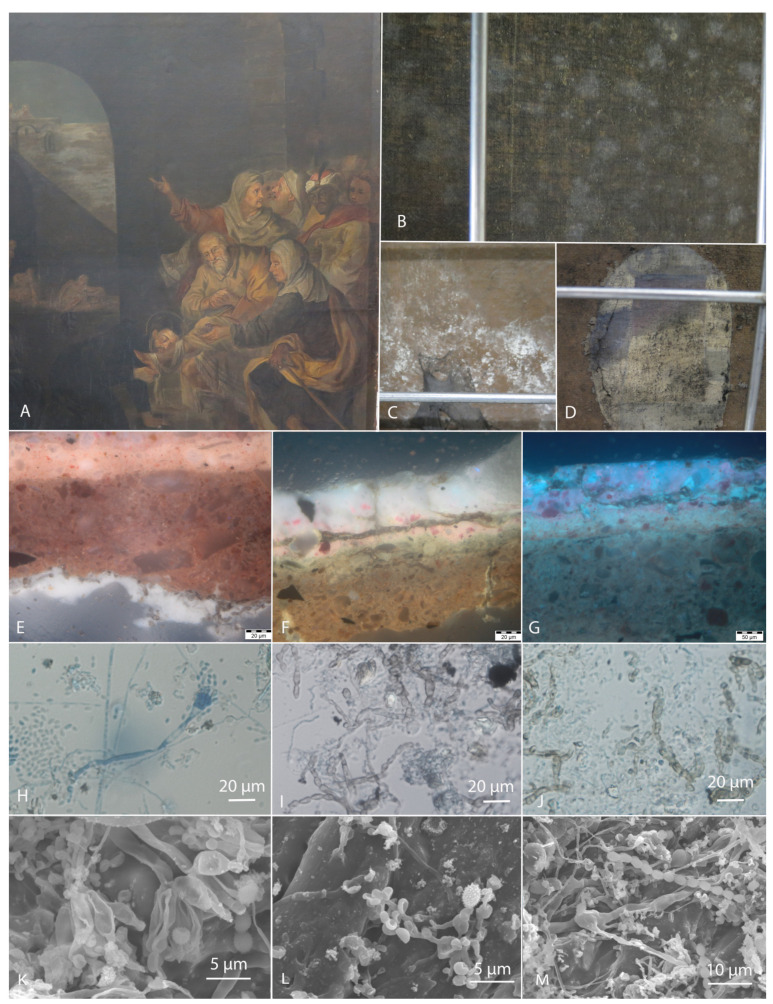
Historic oil painting RCS 15 [37] from the church with signs of biodeterioration on both sides. (**A**) Front side of the painting, (**B**–**D**) Back (courtesy of Polona Zalar), (**E**–**G**) Stratigraphy of the painting under UV light (courtesy of Katja Kavkler) showing the isolation layer between the canvas and the ground (**E**), the ground layer, the paint layer, and the surface of the varnish (**F**), and the ground layer and the paint layer (**G**), all with visible penetration of dark septate hyphae. Light (**H**–**J**) (courtesy of Polona Zalar) and Scanning electron microscopy (SEM) (**K**–**M**) images (courtesy of Petra Bešlagić), show conidiogenous structures of *Aspergillus* on the back (**H**,**K**) and massive growth on the front side of the painting (**I**,**J**,**L**,**M**).

Fungi contaminate both the front (verso) and the back (recto) of canvas paintings (Figure 1). Some studies suggest that front side communities of oil paintings are inactive, while those observed on the back side are considered to be involved in biodeterioration [19]. The infestation on the back side is initiated by the hydration of the organic textile fibres [20], the weakening of the adjacent layers, and the mechanical penetration of the hyphae into the paint layer, resulting in cracks [7,21], considered as hotspots for fungal growth [22]. On the other hand, infestation on the front side depends on the ground, paint, and varnish layers and their mode of application. After gravitational deposition of transient airborne bacterial and fungal spores on the surface, they form a subaerial community that eventually grows and degrades these materials, especially in the presence of high humidity [19].

Although species of the genera *Penicillium*, *Aspergillus*, *Alternaria*, *Cladosporium,* and *Chaetomium* dominate the fungal communities on canvas paintings, their biodeterioration potential is not yet fully known due to the paucity of studies [3,11,14,15]. Moreover, these genera contain numerous species [23,24,25,26,27], and new species are constantly being described [27], making it difficult to identify them correctly.

Canvas and protein degradation are the most frequently studied deterioration activities attributed to fungi contaminating paintings. Representatives of the genera mentioned above [7] are known for their ability to disrupt cellulose fibres by affecting the degree of polymerization, chain length, crystallinity, and orientation, thus increasing their susceptibility to biodegradation by cellulolytic enzymes. Enzymes involved in the degradation of textiles are cellulases (endoglucanases, β-glucosidases) [28,29], hemicellulases, lignin-degrading enzymes, and amylases if the canvas is sized with starch or gelatinases if it is sized with animal glues [30]. The degradation of canvas support allows the fungal hyphae to reach the paint layer, which can lead to delamination, cracking, and discoloration [7]. Depending on the type of paint (oil, tempera, synthetic), different binders are used, such as linseed oil, egg yolk, egg white, casein, vegetable oils and gums, wax, and natural or synthetic resins [31]. Casein and egg tempera are the most susceptible to microbial attack, followed by emulsion distemper (greasy tempera), while linseed oil (drying oil) is the most resistant [7]. The proteolytic activity of fungi is widespread and particularly dangerous for damaging natural binders and materials used in conservation processes [32]. Varnishes protect the painted surface. In traditional painting, they are usually applied after dissolving natural resins in oil, such as vernice comune (mixture of colophony/oleoresin and linseed oil), or by dissolving terpenoid resins in spirits of wine and turpentine [33]. There are rare reports of biodegradation of natural resins by bacteria and fungi [34,35,36].

The present study aims to describe the deterioration potential of xerotolerant/xerophilic and halotolerant/halophilic fungi, which are adapted to dry environments and low water activity (a_w_) of substrates and were recently reported from historic canvas paintings in Slovenia (Figure 1) [22,37]. The most abundant genus was *Aspergillus*, with numerous obligate xerophilic species not previously observed on contaminated paintings. Xerophilic/xerotolerant *Wallemia*, *Penicillium,* and *Cladosporium* species were also abundant. Machine learning predictive models, based on the collected data on materials and contaminants, indicated that different *Aspergillus* species could be associated with all of the materials constituting the paintings [22], in line with the diverse enzymatic activities described in this genus [7,14]. The exact biodeterioration potential, especially of the newly discovered obligate xerophilic species, is not yet known. These fungi have been almost completely neglected in most studies on the deterioration of cultural heritage objects, including canvas paintings, due to the general assumption that low humidity environments prevent fungal growth [22,38]. In this study, 55 fungal strains belonging to 46 species from 16 genera, including 12 obligately xerophilic *Aspergillus* and *Wallemia* strains, were evaluated for their biodeterioration abilities. Acidification of the substrate was tested in two ways: (i) in a liquid medium allowing pH measurement after incubation, and (ii) on a solid medium containing calcium carbonate (CaCO_3_), which dissolves when the medium is acidified [32,39,40]. The strains were screened for the following enzymatic activities, which were selected according to the substrates available in paintings: cellulases (endocellulase, β-glucosidase), amylase, and laccase, which cause canvas degradation, gelatinase and caseinase which cause protein degradation, while esterase and lipase-lecithinase cause degradation of drying oil. The production of fungal pigments, which is dependent on the substrate, was recorded on all media tested that did not contain indicator dyes or reagents that cause colour reactions. Several tests were used for the first time to assess painting biodeterioration: the production of enzymes laccase, urease, and DNase, the formation of siderophores, and the utilization of aromatic and aliphatic sole carbon sources. Laccase, an enzyme known for lignin degradation, can also oxidize other materials containing aromatic rings (e.g., as components of pigments and modern synthetic materials) [41]. Urease can act on several materials in paintings, as urea can be used as a dissolving agent for powdered dyes [1,7] or in combination with esterase can assist in the degradation of wax and synthetic resins [42]. A DNase assay was performed to evaluate the ability of fungi to hydrolyze DNA from surface, formerly active microorganisms and use it as a source of carbon and energy for growth [1]. The siderophore production tested allows the recruitment of scarce Fe from the environment [43,44,45]. The ability to degrade aromatic and aliphatic substrates was used to target the potential degradation of synthetic restoration materials containing aromatic rings or aliphatic chains in their structure, such as the hot-sealing adhesive BEVA 371 and the acrylic glue Lascaux 498 HV, respectively. While the enzyme screening of the xerotolerant and moderately xerophilic fungi was carried out according to the established culture media and associated protocols, all culture media were adapted to test the enzyme production of the obligate xerophilic fungi by a_w_ reduction with the addition of glycerol. Based on the results obtained, we conclude that the fungal taxa tested are hazardous to paintings, obligately xerophilic ones, even when stored in depositories with recommended environmental measures.

## 2. Materials and Methods

### 2.1. Fungal Strains and Inocula Preparation

Identified fungal strains were selected from the studies of Kavkler et al. (2022) [37] and Zalar et al. (2023) [22]. They were mainly isolated from deteriorated historic paintings exhibited in churches or were deposited for restoration in authorised depository (Supplementary Table S1). Only two strains originate from the air in the painting depository of the Restoration Centre of the Institute for the Protection of Cultural Heritage of Slovenia (RCS). Four strains were isolated as contaminants of mock-up samples treated with restoration materials. The later were identified using DNA sequences of genus-specific molecular markers evident in Supplementary Table S1 as described in Zalar et al. (2023) [22]. Nucleotide sequences were deposited to NCBI (PP033021, PP034128, PP034131, PP034132). All the strains are preserved in the Microbial Culture Collection Ex within Infrastructural Centre Mycosmo (Department of Biology, Biotechnical Faculty, University of Ljubljana, Ljubljana, Slovenia).

Fungal cultures were grown on Malt Extract Agar (MEA) prepared according to Galloway and Burgess (1952) (0.02% malt extract, 0.02% glucose, 0.001% pepton, 0.02% (m/V) agar; pH = 5–5.5) [46] or on Dichloran Glycerol Agar [47] (DG18; Biolife Italiana, Milano, Italy) prepared according to the producer’s instructions, for 7–14 days. Conidia/spore suspensions of sporulating fungi were prepared with concentration of 10^7^ spores/mL, while suspensions of mycelial fragments of nonsporulating fungi were not standardized. The inocula of xerotolerant and moderately xerophilic species were prepared in 0.9% sodium chloride (NaCl), while those of obligate xerophiles were prepared in 10% NaCl.

### 2.2. Growth at Different Water Activities and Temperatures

All selected strains (see Section 2.1) were tested for growth on media without and with added solutes (glycerol, NaCl) to determine their xero-(halo)tolerance/xero-(halo)philic nature. Xerotolerance was tested on DG18 culture medium base (Biolife Italiana, Milano, Italy) [47] without (DG0; a_w_ = 0.99) and with the addition of different amounts of glycerol: 18% (DG18; a_w_ = 0.942), 30% (DG30; a_w_ = 0.882), and 40% (*w*/*v*) (DG40; a_w_ = 0.827). Halotolerance test was performed on MEA (described in 2.1) supplemented with different concentrations of NaCl: 10% (*w*/*v*) (MEA10; a_w_ = 0.930), 20% (MEA20; a_w_ = 0.852), and 25% (MEA25; a_w_ = 0.802). Colony diameters were measured for both tests after 28 days of incubation at 24 °C. Water activity of the media was measured using AquaLab Series 3TE (Decagon Devices, Pullman, WA, USA).

Strains were tested for growth at 15 °C (recommended temperature in depositories), 24 °C (museum temperature), and 37 °C (temperature used to test potential hazards to human [48]) on MEA for xerotolerant and moderately xerophilic fungi, or on DG18 for obligately xerophilic species. Colony diameters were measured after 14 days using a ruler.

### 2.3. Biodegradation Assays

#### 2.3.1. Excretion of Enzymes

**Figure 2:**
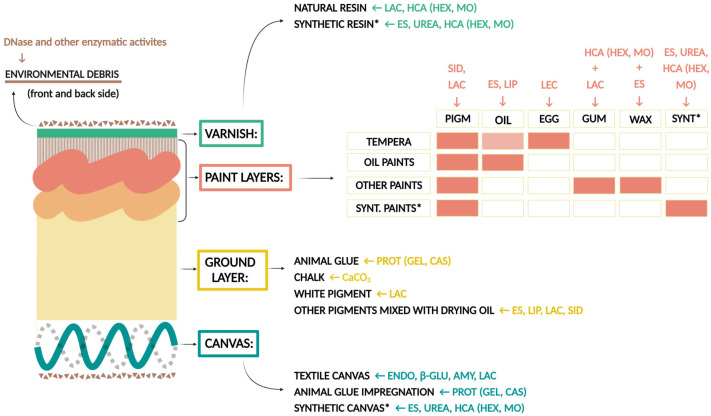
Graphical visualization of the selected enzyme tests according to the materials used in painting layers. AMY: amylase; ENDO: endocellulase; β-GLU: β-glucosidase; PROT: proteinase activity (CAS: caseinase; GEL: gelatinase); ES: esterase, LIP: lipase; LEC: lecithinase; LAC: laccase, CaCO_3_: carbonate dissolution; UREA: urease; HCA: hydrocarbon assimilation (HEX: hexadecane, MO: mineral oil); SID: siderophore formation; PIGM: pigments; DRY: drying oil; EGG: egg; GUM: gum; WAX: wax; SYNT: synthetic materials; * contemporary techniques using synthetic materials. (Created with BioRender.com).

Enzyme screening assays were selected based on the commonly used materials in canvas paintings and in their conservation–restoration. The selection according to a specific painting layer is presented in Figure 2. The composition of paint layers according to the painting technique (egg tempera, oil paints, other paints, or synthetic paints) is indicated with orange rectangles, e.g., greasy tempera contains pigments, drying oil, and egg. Each component can be degraded by specific enzymes, e.g., pigments by laccases, drying oil by esterases and lipases, and egg by lecithinases. The potential hazard of one enzymatic activity may affect multiple layers of a painting, as, e.g., in the case of esterase activity.

The following enzyme activities were tested on agar media in 9 cm Petri dishes: amylase, β-glucosidase, endocellulase, protease (caseinase as general activity, gelatinase as specific activity), esterase, lecithinase, lipase, and laccase. Media were 3-point inoculated with 5 µL of conidial suspension and incubated at 24 °C for 7–28 days. The deterioration profile of xerotolerant and moderately xerophilic taxa was tested on established culture media, as described in detail below, while for the testing of obligately xerophilic fungi, the water activity (a_w_) of all test media was adjusted to 0.94 by adding 18% (*w*/*v*) glycerol. Agar media for lecithinase activity was the only one prepared without additional solutes, but it was incubated for a longer period (28 days) until sufficiently dried to allow the growth of obligately xerophilic species.

The overview of performed enzyme tests, the main substrate, and positive results is presented in Table 1, while the exact composition and preparation of the test media are described below.

Amylolytic activity was tested for degradation of starch used as a canvas consolidant [1,7]. The medium used was Czapek agar, prepared from starch solution (5% soluble starch in dH_2_O) (Sigma-Aldrich, Steinheim, Germany), 50 mL of Czapek solution A (4% NaNO_3_, 1% KCl, 0.02% MgSO_4_ × 7H_2_O in dH_2_O), 50 mL of Czapek solution C (2% K_2_HPO_4_ in dH_2_O), 1 mL of zinc solution (1% ZnSO_4_ × 7H_2_O in dH_2_O), 1 mL of copper solution (0.5% CuSO_4_ × 5H_2_O in dH_2_O), 1.2% agar, and dH_2_O up to 1 L. After 14 days of incubation, plates were flooded with iodine solution for 30 s, and the excess iodine was poured off; the clear zone around colonies in otherwise black/blue-stained medium indicated starch degradation [49].

Endocellulase and β-glucosidase activities were tested as indicators for canvas degradation [1,7]. To test β-glucosidase activity, aesculin (6,7-dihydroxycoumarin 6-glucoside) (Merck, Darmstadt, Germany) was added to Czapek agar at the concentration 0.3%; after 14 days of incubation, a black zone around colonies indicated a positive reaction. Endocellulase activity was tested by adding 4% carboxymethyl cellulose (CMC; Sigma-Aldrich, Steinheim, Germany) to Czapek agar; after 21 days of incubation, media were stained with 0.3% Congo red (Merck, Darmstadt, Germany) and rinsed after 30 min with 1 M NaCl solution; a yellow clear zone around colonies indicated a positive reaction [49].

Caseinase activity was tested as the general proteolytic activity [1,7] on agar medium containing 5% skim milk (Merck, Darmstadt, Germany) [50]. The medium was prepared from two separately prepared and autoclaved solutions: (1) 0.2% yeast extract, 4% agar in dH_2_O; (2) 10% skim milk in dH_2_O. After autoclaving, 500 mL of solution (1) was aseptically and homogeneously added to the same amount of solution (2); a clear zone in an otherwise opaque medium indicated proteolytic activity which was read after 14 days.

Degradation of gelatine, a specific protein often used as a sizing agent to alter the absorbent qualities of canvas [1,7], was tested on Czapek agar medium described above with addition of 1% sucrose and 12% gelatine (Sigma-Aldrich, Steinheim, Germany) in 1 L of dH_2_O. After 14 days of incubation, a positive reaction resulted in liquefied medium around colony [49].

Esterase, lecithinase, and lipase were tested, because they can be involved in the drying oil degradation [1,7]. Esterolytic activity was determined on an agar medium containing 1% Tween-80 (polyoxyethylene-sorbitan-monooleate) (Sigma-Aldrich Steinheim, Germany), 1% peptone, 0.5% NaCl, 0.01% CaCl_2_ × 2H_2_O, 1.5% agar, and 0.0025% pH indicator Bromocresol purple (Sigma-Aldrich, Steinheim, Germany) with final pH of 5.4 [51]; the increase in pH due to cleavage of ester bonds resulted in a colour change of the pH indicator, switching from yellow to purple/blue, and in precipitation of calcium salts around the colonies; test was read after 14 days. Lecithinase activity (esterolytic) was determined on egg yolk agar that containing 1.5% pancreatic digest of casein (Sigma-Aldrich, Steinheim, Germany), 1% vitamin K_1_, 0.5% NaCl, 0.5% papaic digest of soybean meal, 0.5% yeast extract, 0.04% L-cystine, 0.5% hemin, 10% egg yolk emulsion, 2% agar in dH_2_O, pH 7.0 [52]; a positive reaction was evident when a white opaque zone formed around the colonies; the reaction was read after 28 days. Lipase activity (esterolytic) was assayed on medium containing 0.5% mycological peptone, 0.3% yeast extract, and 1% agar. After autoclaving and cooling to 60 °C, filter sterilized tributyrin (Sigma-Aldrich, Steinheim, Germany) was added to the final concentration of 0.1% [53]; clearing of the opaque medium around the colonies indicated positive reaction, read after 28 days.

Ureolytic activity was tested, because urea can be used as dissolving agent for powdered dyes [1,7]. Urease in combination with esterase supports the degradation of wax and synthetic resins [42]. The testing medium was prepared in two steps: Medium containing 0.1% peptone, 0.1% glucose, 0.5% NaCl, 0.2% KH_2_PO_4_, 0.0012% phenol red (Sigma-Aldrich, Steinheim, Germany), 2% agar in dH_2_O, pH 6.8 to 7.0, was autoclaved. After cooling, filter-sterilized urea solution (Merck, Darmstadt, Germany) was added to the final concentration of 2% [54]. Medium was aseptically poured into sterile tubes and cooled down in a slanted position. After 14 days, ureolytic strains increased pH of the medium due to urea degradation, visible as change in pH indicator colour from yellow to purple.

Laccase (ligninolytic) activity was tested for lignin degradation either in textile (jute, hemp or linen) or mainly in wooden support or non-specific oxidative action on resins, varnishes, and pigments [1,7]. Laccase (ligninolytic) activity was tested on potato dextrose agar (PDA) (Biolife Italiana, Milano, Italy) [55] containing either 0.0015% (*m*/*v*) ABTS (2-2′-azino-bis-[3-ethyl benzthiazoline-6-sulfonic acid]) (Sigma-Aldrich, Steinheim, Germany) or 0.0005% (*m*/*v*) guaiacol (Sigma-Aldrich, Steinheim, Germany) as separate substrates; purple and orange/brown colour around colonies, respectively, indicated laccase activity.

The ability to hydrolyse DNA was tested to assess the ability of fungi to utilize DNA as the residues of microbes deposited in the dust or present on the surface (see Section 2.3) [1,7]. DNase agar [56] (Becton and Dickinson, Franklin Lakes, NJ, USA) was prepared according to the manufacturer’s instructions; after 7 days of incubation, plates were flooded with 1 M HCl; a change in the medium from an opaque white colour to a transparent, indicated a positive reaction.

#### 2.3.2. Substrate Acidification

Acidification due to the growth of fungi can cause depigmentation of canvas paintings and alter chemical composition of the materials [11,14,29]. To determine acidification properties of the selected isolates, we used two tests: (i) Modified protocol of Borrego et al. (2010) [57]: Minimal liquid medium, containing 0.3% NaNO_3_, 0.1% K_2_HPO_4_, 0.05% MgSO_4_ × 7H_2_O, 0.05% KCl, 0.001% FeSO_4_ × 7H_2_O, and 0.1% glucose, was prepared in distilled H_2_O. Ten mL of the medium was aliquoted into 20 mL test tubes and autoclaved. Prior autoclaving-adjusted pH 7 dropped to pH 6 after autoclaving. A loop full of mycelium in case of nonsporulating fungi or 10^5^ of spores was inoculated into the test medium. After 7 days of incubation, the resulting pH was measured using pH indicator strips (Macherey-Nagel, Düren, Germany and Supelco, Bellefonte, PA, USA). Acidification properties were also evaluated by the ability of fungal strains to dissolve calcium carbonate (CaCO_3_), on agar medium prepared according to [58], containing 0.5% CaCO_3_, 1% glucose, 1.5% agar in distilled H_2_O, pH 8.0. The excreted acids may alter CaCO_3_ ground supporting the canvas. After 14 days of incubation at 24 °C, a clear zone around colonies in otherwise opaque medium indicated acid production, which caused calcite dissolution.

#### 2.3.3. Excretion of Soluble Pigments

Microbially produced pigments excreted in paintings pose an aesthetic problem [49]. Production of soluble pigments was recorded on all solid culture media used in this study that did not contain dyes or colour indicators that could possibly interfere with pigment production. These were MEA, MEA + 10, 20, 25% NaCl, DG18, 30, 40, caseinase, gelatinase tests described in Section 2.3. The production of pigments was recorded after 28 days of incubation.

### 2.4. Assimilation of Hydrocarbons

To evaluate the ability of the strains to utilize aliphatic and aromatic substrates, such as synthetic polymers used in conservation–restoration actions for consolidation, lining, or for painting protection, their growth was tested on mineral oil (aliphatic) and hexadecane (aromatic). The selected strains were inoculated into a liquid medium containing yeast nitrogen base (YNB; Becton and Dickinson, Franklin Lakes, NJ, USA) (pH 7.0) and 0.5% (*w*/*v*) NH_4_(SO_4_)_2_ with 20% (*w*/*v*) filter-sterilized mineral oil/hexadecane (Sigma-Aldrich, Steinheim, Germany). The assay included two controls, a negative (non-inoculated YNB) and a positive control (inoculated YNB with 2% (*w*/*v*) glucose). To evaluate the utilization of mineral oil/hexadecane of obligately xerophilic fungi, water activity (a_w_) was decreased to 0.94 by adding 10% (*w*/*v*) NaCl into the medium. The utilization of mineral oil/hexadecane as the sole carbon source was evaluated after 28 days of incubation at 24 °C [59].

### 2.5. Siderophore Formation

To investigate the possibility of Fe^3+^ sequestration from organic pigments, siderophore production was evaluated. The ability of fungal strains to produce siderophores was tested on chrome azurol S (CAS) agar [45]. Medium was mixed from separately prepared and autoclaved solution 1 and solution 2 in ratio 1:9. Solution 1 contained 0.061% (*m*/*v*) CAS (Acros Organics, Geel, Belgium), 10% (*v*/*v*) iron (III) solution [83 µL HCl (37%, 12 M) and 27 mg FeCl_3_ × 6H_2_O in 100 mL MiliQ water], and 0.073% CTAB in 100 mL of dH_2_O. Solution 2 was prepared from 0.034% (*m*/*v*) PIPES, 3.78% NaOH, 0.022% (*m*/*v*) malt extract, 0.001% (*m*/*v*) peptone, 0.022% (*m*/*v*) glucose in 900 mL of dH_2_O. The positive reaction was a colour reaction ranging from blue to orange, purple, or magenta. The plates were observed after 14 days of incubation at 24 °C.

### 2.6. Evaluation and Categorization of Fungal Activities

To allow the comparison among the tested strains, fungi were categorized into groups, or factors were determined according to below described criteria.

Based on the ability to grow on media with different water activities obtained by the addition of glycerol, fungi were categorized into six groups. Group »0« represents non-xerotolerant fungi (growth only in medium without added glycerol). Groups 1–2 indicated xerotolerant fungi, where »1« defines weakly xerotolerant (growth on agar media without and with 18% (*w*/*v*) glycerol), »2« moderately xerotolerant, and »3« moderately xerophilic (growth on all tested agar media). Groups 4 and 5 indicate obligately or extremely xerophilic fungi, where »4« means that fungi required 18% (*w*/*v*) glycerol for growth, and »5« defined fungi that grow only in the presence of 30 and 40% (*w*/*v*) glycerol.

Based on their ability to grow on agar media with different concentrations of NaCl, fungi were categorized into six groups: »0« non-halotolerant (no growth on media supplemented with 10% NaCl); »1« weakly halotolerant (growth without and with 10% NaCl); »2« moderately halotolerant (growth without and with maximally 15% NaCl); and »3« moderately halophilic (growth at 10, 20, and 25% NaCl). Groups 4 and 5 indicate obligately or extremely halophilic fungi, where »4« represents fungi growing only in the presence of 10 or 15% NaCl, and »5« is fungi growing at minimally 15% NaCl.

According to the growth at different temperatures, fungi were categorized into four groups: »1« psychrophile (growth only at 15 °C); »2« psychrotolerant (growth at 15 and 24 °C); »3« psychrotolerant mesophile (growth at 15, 24, 37 °C); and »4« mesophile (growth at 24 and 37 °C).

To allow the comparison of enzymatic activities between the tested strains, results were evaluated, when possible, as enzyme coefficients according to the following adapted equation [60]: F = diameter of the coloration zone/colony diameter. Enzymatic activity factors (relative values of enzymatic activity) were determined based on the range of calculated enzymatic activity coefficients. Coefficients equal to or greater than 1 were divided into five equal activity classes, where »1« represented the lowest and »5« the highest activity. The most extreme values were excluded when setting the criteria. Lecithinase activity and urease activity were scored as positive/growth (1) or negative reaction/no growth (0).

According to the measured pH after incubation, fungi were categorized into three groups: »0« when final pH remained 6.0–6.1; »1« when final pH was 5.0–5.9, and »2« when final pH was reduced to 4.0–4.9. Ability to solubilize calcium carbonate (CaCO_3_) was scored with three categories; »0« negative reaction (no solubilization of CaCO_3_); »1« weak solubilization; and »2« strong solubilization. Production of soluble pigments was scored as positive »1« (pigmentation of the medium) or negative »0« (no pigmentation).

Assimilation of hydrocarbons was scored as positive, where »1« represents weak growth, »2« very good growth, and »0« as negative or no growth. Siderophore formation was scored the same as enzymatic activities, with final factors from 0 to 5.

### 2.7. Statistical Analyses

To investigate the possible differences between the enzymatic profiles of xerotolerant and moderately xerophilic, and xerophilic species, a table of the presence and absence of specific enzymatic activities of the tested fungal strains as well as their ability to change (reduce) pH and pigmentation was prepared visually as described below. The final figures were prepared by assembling the individual panels in Inkscape [61].

Fungal isolates were clustered with Principal Component Analysis (PCA), Multidimensional Scaling (MDS), and UMAP, Uniform Manifold Approximation and Projection (UMAP). Analyses were performed in the environment R [62] using the function ‘prcomp()’ (PCA) and the function ‘cmdscale()’, both from the package ‘stats’ (MDS), and the function ‘umap()’ from the package ‘umap’ [63]. A table of species and their enzymatic activities was used as input. The ellipses surrounding groups of strains were added with the ‘stat_ellipse()’ function of ‘ggplot2’ [64].

The presence and absence of specific enzymatic activities in tested strains as well as selected strain characteristics (pH-change, xerophily/halophily, pigmentation) were visualised with UpSet plots in the environment R [62] using the package ‘ggplot2’ [64].

## 3. Results

### 3.1. Physiological Characterisation: Growth at Different Water Activities, Temperatures, and Production of Acids and Pigments

**Figure 3:**
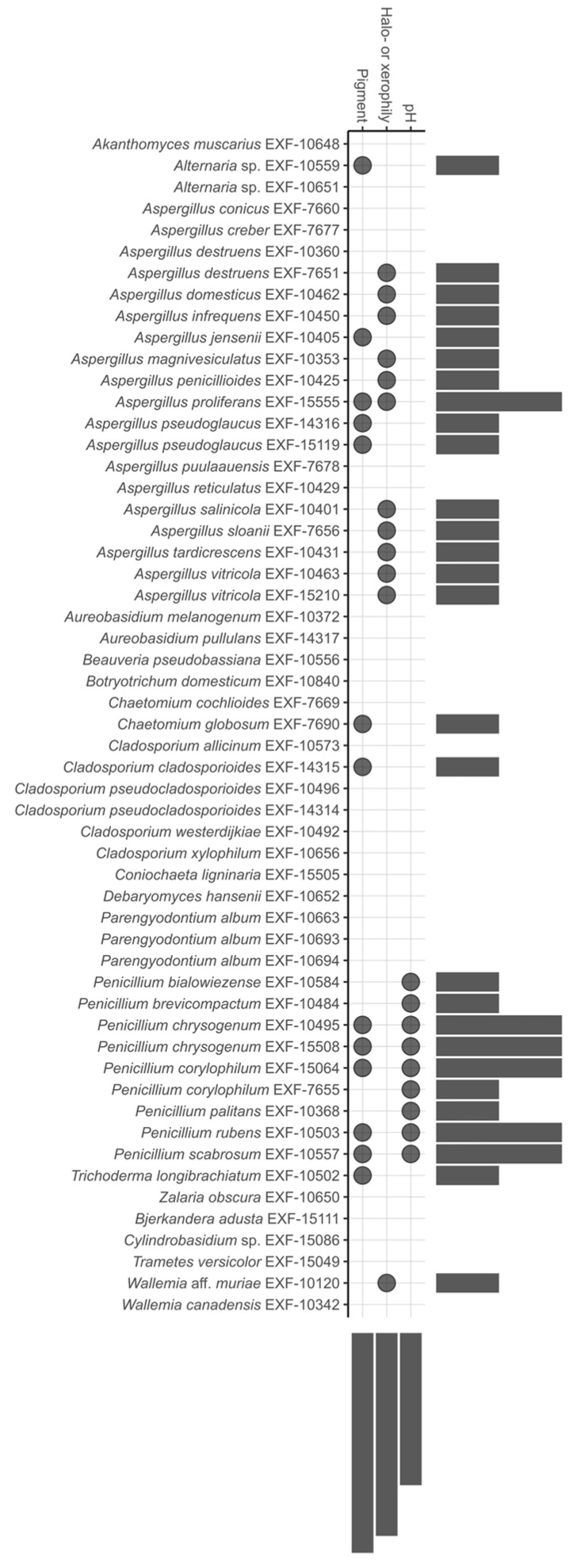
The UpSet plot [65] listing fungi showing selected features under in-vitro conditions that, if performed also on canvas paintings, could have a damaging effect, like acidifying abilities (change of pH from 6 to 5.1), growth at extreme a_w_ conditions (obligate halo- or xerophily), and soluble pigment production. Dots represent positive character expressions of a specified taxon and horizontally aligned bars the number of expressed characters (0–2). Vertically arranged bars specify the number of taxa expressing a certain character in relative terms. The figure was generated by ‘ggplot2’ [64].

Figure 3 provides an overview of the three characteristics studied: xerophily, significant acidification of the liquid medium (from pH 6 to pH 5.1), and soluble pigment production, each of which is represented by a dot. More detailed results and additional data on growth at different temperatures are summarized in Supplementary Table S2.

More than one third of all tested strains, mostly species of the genus *Aspergillus* and *Wallemia* aff. *muriae*, showed an obligate need for low water activity of the medium, regardless of the solute and could thus be designated as obligate or extreme xerophiles or halophiles (17.5%). If no preference was evident for NaCl in comparison to glycerol, they were categorized as extreme xerophiles. The only species preferring NaCl over glycerol was *A. proliferans*. Differences were observed between *A. destruens* strains, with one being obligately xerophilic and the other only tolerant.

**Figure 4:**
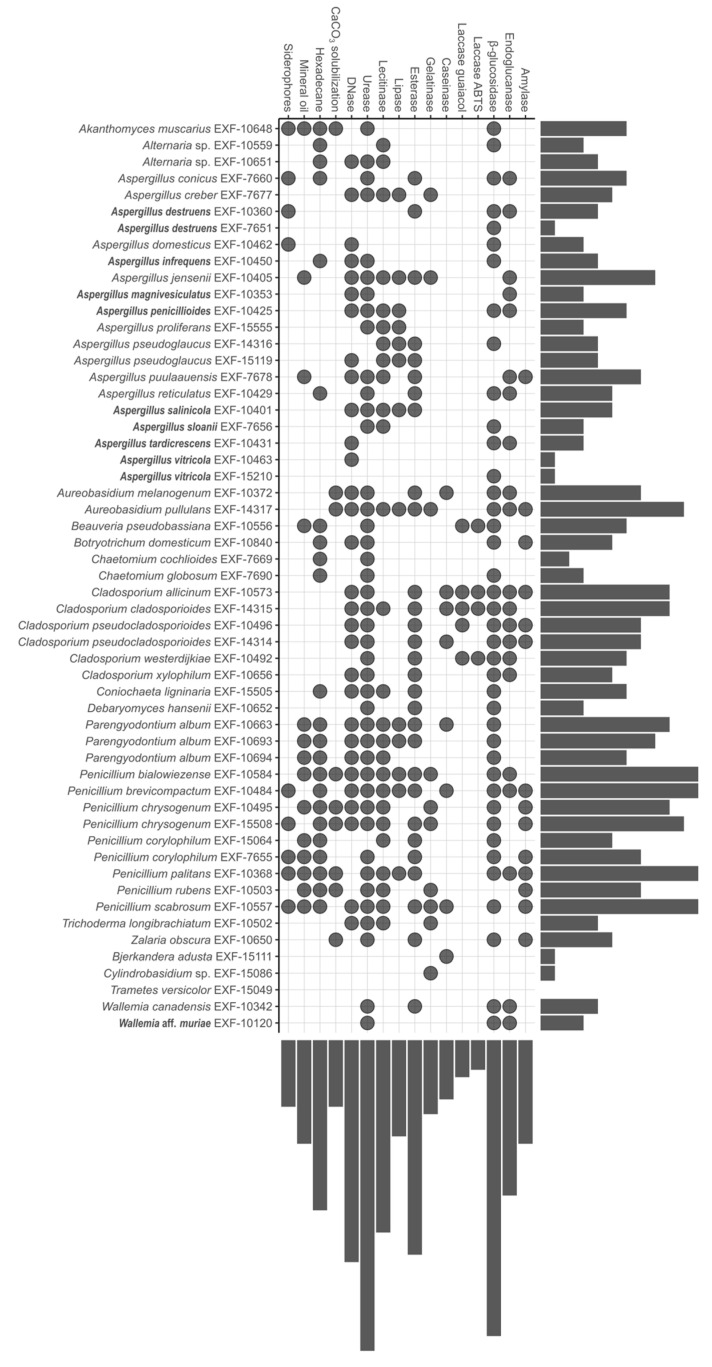
The UpSet plot [65] shows strong activities (factor 2 or higher) of the tested fungal strains, presented in an alphabetical order of ascomycetes, continuing by alphabetical order of basidiomycetes, from top to bottom, at the left side of the plot. Beside enzyme activities (amylase, endoglucanase, β-glucosidase, laccase, caseinase, gelatinase, esterase, lipase, lecithinase, urease, and DNase), the ability to solubilize CaCO_3_, growth on hexadecane and mineral oil as sole carbon sources, and siderophore production are shown. Obligately xerophilic taxa are written in bold. Dots represent positive character expressions of a specified taxon and horizontally aligned bars the number of expressed characters (0–11). Vertically arranged bars specify the number of taxa expressing a certain character in relative terms. The figure was generated by ‘ggplot2’ [64].

All strains except for *Penicillium* were able to maintain pH 6. Most *Penicillium* strains substantially lowered the pH of the medium: *P. bialowiezense*, *P. brevicompactum*, *P. corylophilum* and *P. rubens* for 0.9–1 pH unit, and *P. chrysogenum*, *P. palitans* and *P. scabrosum* for 1.1–2 pH units (Supplementary Table S2). Although, the CaCO_3_ solubilization test (implemented in Figure 4) showed that an even smaller pH drop resulted in a positive result, evident in many aspergilli and some other fungi (Supplementary Table S3).

Fifteen strains, mostly penicillia, were able to excrete different pigments observed on at least one of the culture media viewed.

Almost all tested strains were able to grow at temperatures recommended for storage and exposition of canvas paintings (Supplementary Table S2). In total, 3 strains grew only at 15 °C and were identified as psychrophiles, 31 strains were psychrotolerant, and 19 strains were psychrotolerant mesophiles and grew at all tested incubation temperatures (15, 24, and 37 °C).

### 3.2. Biodeteriorative Potential of Fungi: Enzymatic Activities, Ability to Assimilate Hydrocarbons, to Solubilize CaCO_3,_ and Synthesize Siderophores

An overview of the strains that displayed the strongest enzymatic activities or strong ability to assimilate hydrocarbons, solubilize CaCO_3_, and sequester iron by producing siderophores (factor 2 or higher) is shown in Figure 4. Detailed results are summarized in Supplementary Table S3, and the majority of the tests are presented in Supplementary Figure S1.

Strains that displayed the largest number of strong activities, 11 strong activities out of 16, belong to different xerotolerant or moderately xerophilic *Penicillium* species: *P. bialowiezense*, *P. brevicompactum*, *P. palitans* and *P. scabrosum*, and *Penicillium chrysogenum* (EXF-15508), while *Aureobasidium pullulans* displayed 10 strong activities. Three strains showed a single strong activity: *A. destruens* (EXF-7651) β-glucosidase, *A. vitricola* (EXF-10463 DNase), *Bjerkandera adusta* caseinase, and *Cylindrobasidium* sp. gelatinase, while *Trametes versicolor* demonstrated no strong activities.

#### 3.2.1. Enzymatic Activity

The majority of xerotolerant and moderately xerophilic strains showed a strong urease activity (80.0%), and less strains demonstrated β-glucosidase (68.9%), esterase (62.2%), DNase, and lecithinase (both 51.1%) activities. Endoglucanase, amylase, lipase, gelatinase, caseinase, and laccase were displayed as weak reactions. *Penicillium brevicompactum* displayed almost all strong enzymatic activities and represents the most active and enzymatically diverse species tested, followed by *Aureobasidium pullulans*, *Cladosporium allicinum,* and *C. cladosporioides*. The least enzymatically active xerotolerant or moderately xerophilic fungi were *Bjerkandera adusta*, *Chaetomium cochlioides*, and *Cylindrobasidium* sp. With a single activity, while drought sensitive *Trametes versicolor* did not display any of the tested activities.

Among obligately xerophilic isolates, more than two thirds showed β-glucosidase activity (75%), and more than half expressed DNase (58.3%) and urease (50.0%). *Aspergillus penicillioides* displayed five and *A. salinicola* six strong enzymatic activities. As mentioned above, only one of two tested *A. destruens* (EXF-10360) and *A. vitricola* (EXF-10463) strains showed a single strong enzymatic activity, β-glucosidase and DNA-se, respectively.

In comparison, more than a half of the obligately xerophilic strains showed strong β-glucosidase, DNase, and urease activities, while more than half of the xerotolerant and moderately xerophilic strains displayed strong esterase and lecithinase activities. The frequency of the strong enzymatic activities was at least 16.7% among obligately xerophilic fungi, excluding gelatinase, caseinase, laccase, and amylase activities that were not recorded. Only weak amylase activity was present among obligately xerophilic strains. In contrast, among xerotolerant and moderately xerophilic fungi, all of the strong enzymatic activities were detected in at least 20% of the strains, excluding laccase activity that was less frequent. In conclusion, this group of fungi displayed more diverse and stronger enzymatic activities.

#### 3.2.2. Assimilation of Hydrocarbons, Solubilization of CaCO_3,_ and Production of Siderophores

In general, 42.1% of all tested strains were able to assimilate hexadecane, while other strong activities were less frequent. Xerotolerant and moderately xerophilic strains were able to assimilate hexadecane (51.1%) in an even higher proportion, while other activities were less common. Two strains strongly displayed all four activities, *Akanthomyces muscarius* and *P. palitans*, while moderately xerophilic *P. bialowiezense*, both strains of *P. chrysogenum*, *P. corylophilum* (EXF-7655), *P. rubens,* and *P. scabrosum* displayed at least three strong activities of four tested. Almost 40% of xerotolerant and moderately xerophilic strains did not show any of these activities.

A strong ability to synthesize siderophores and an ability to assimilate hexadecane were detected as well among obligately xerophilic strains, however in less than 20%, while none of them were able to assimilate mineral oil and to solubilize CaCO_3_. Four strains out of twelve showed only one strong activity. *A. destruens* (EXF-10360) and *A. domesticus* both produced siderophores, *A. infrequens* grew abundantly on hexadecane, and *A. conicus*, *A. domesticus,* and *A. destruens* produced siderophores. Other obligately xerophilic strains did not display any of these activities.

The comparison of xerotolerant and moderately xerophilic fungi to obligately xerophilic fungi showed that, in general, the former were able to solubilize CaCO_3_ to assimilate hydrocarbons, particularly hexadecane (51.1%), and synthesize siderophores. In contrast, obligately xerophilic fungi only assimilated hexadecane and synthesized siderophores (16%).

#### 3.2.3. Statistical Analyses

**Figure 5:**
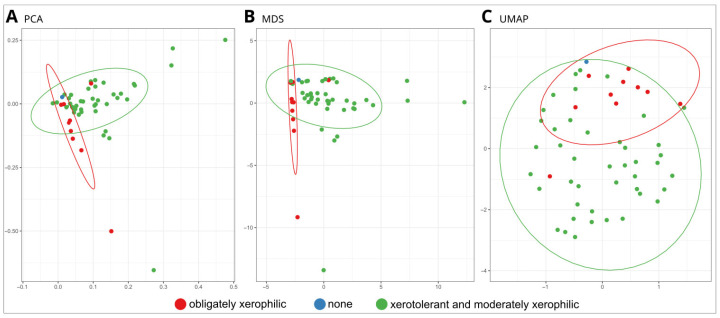
Comparison of dimensionality reduction results based on the presence/absence of enzymatic activities, pigmentation, and pH reduction. (**A**) principal components analysis (PCA); (**B**) multidimensional scaling (MDS); and (**C**) Uniform Manifold Approximation and Projection (UMAP). Each individual strain is labelled with a dot, and groups are marked using colour: obligate xerophiles in red, xerotolerant and moderately xerophilic in green, non-xerotolerant in blue.

The phenotypic traits of tested fungal strains (the presence of different enzymatic activities, the presence of pigmentation, and change in pH) were distinctly different between obligately xerophilic and xerotolerant and moderately xerophilic strains. Dimensionality-reduction methods clustered obligately xerophilic strains into a relatively tight cluster that was only partially overlapping with a larger cluster of xerotolerant and moderately xerophilic strains in both PCA and MDS analyses and to a somewhat lesser extent using UMAP (Figure 5).

## 4. Discussion

Canvas paintings are objects susceptible to biodeterioration, because they are rich in organic and inorganic materials that provide food and support for fungi under favourable environmental conditions [1]. Fungi are ubiquitous, and their propagules are constantly present in the air and dust, so inoculum is constantly available [66], but environmental conditions control their growth, particularly RH. Studies of biodeteriorated paintings usually focus on biodiversity [3,67], to a lesser extent on the detection of viable taxa, e.g., the presence of active mycelium, and even less on the biodeterioration potential in terms of enzyme and acid excretion [11,14,32]. The aim of this study was to investigate the biodeterioration potential of xerophilic species likely to be active in low humidity environments.

### 4.1. Xerophily Is the Crucial Factor That Allows the Biodeterioration of Paintings Indoors

Although definitions of xerophily can vary from publication to publication, Pitt (2008) defined xerophile as a fungus capable of growing under at least one set of conditions with a water activity of 0.85 or less [68]. In our study, this a_w_ was only achieved on DG40 (a_w_ = 0.827), MEA + 20% NaCl (a_w_ = 0.852), and MEA + 25% NaCl (a_w_ = 0.802), which means that all fungi classified as groups 3–5 on the glycerol and NaCl media can be considered as xerophiles. For practical use, we distinguish obligate xerophiles from moderate xerophiles, with the former requiring a reduction in a_w_ for growth.

RH and T are recognised as risk factors in museums/storage facilities and are therefore maintained at 40–60% RH with 5–10% variation over 24 h and in the T range of 16–25 °C [69]. Nevertheless, fungal growth may occur due to local RH increases in the indoor microclimate, e.g., near walls or in poorly ventilated areas [2,70,71], allowing the growth of xerotolerant and xerophilic taxa. However, xerophilic fungi with a wide a_w_ range can grow under the recommended environmental regime and cause biodeterioration. This phenomenon is partly due to their ability to accumulate and retain extraordinary amounts of glycerol for osmotic adjustment [72]. In recent years, more studies have pointed to xerophilic fungal communities that are not constrained by the above mentioned environmental parameters [38] and to their as yet unassessed biodegradation potential [22].

Among the limited number of fungal genera with xerophilic species, the genus *Aspergillus* (Ascomycota, Eurotiales) is one of the most common on paintings and contains several groups of xerophilic species restricted to the subgenus *Aspergillus*. While it was generally known that metabolic activity and cell division of the most extreme xerophilic aspergilli cease between a_w_ 0.700 and 0.640 (about 70–64% RH), *A. penicillioides*, the most xerophilic species of the section *Restricti* [23], germinated even at a_w_ 0.640 when culture media were supplemented with glycerol. Furthermore, extrapolations made for this species indicated a theoretical water activity minimum at a_w_ 0.570, 57% RH [72], which is already in the range of recommended RH for museums/depositories. Half of the species accommodated to the section *Restricti* have been recently described as new species [27], also as painting contaminants [22], detected in high CFU numbers. They differ slightly in xerophily, but the majority of species cannot grow without a minimum of 5% NaCl, with an optimum around 10% and a maximum at 20% NaCl [27]. The exact experiments on the limiting a_w_ values for their germination and growth have not yet been studied, but it is quite possible that they are also among the most extreme. Tolerance to low a_w_ is also known in *Penicillium*, e.g., the most extreme species *P. chrysogenum* was observed to germinate at a_w_ 0.78 [68]. Among *Cladosporium* isolates, the *C. sphaerospermum* species complex, in particular *C. sphaerospermum* and *C. halotolerans*, are the most extreme, growing at a_w_ ≥ 0.82, while species of other species complexes have an a_w_ minimum of 0.85 [73]. Basidiomycetes in general are less known from low a_w_ environments, the only exception being *Wallemia*, which germinates at a_w_ 0.75 [68]. In terms of environmental parameters, particularly RH, *Aspergillus* and *Penicillium* are among the most extreme and are likely to be active on paintings. 

### 4.2. Testing the Biodeterioration Potential of Obligate Xerophiles by Adapting Culture Media

Biodeterioration tests performed on painting contaminants in previous studies [22] were carried out on culture media with a_w_ close to 1, which had to be reduced to allow testing of obligately xerophilic species unable to grow without additional solutes. We used the same amounts of glycerol as in dichloran glycerol agar (DG18), suitable for the enumeration and isolation of moderately xerophilic fungi [68], to obtain a_w_ close to 0.942, which is much higher than the recommended indoor RH conditions but allows for relatively rapid growth, in up to 28 days. However, this a_w_ was not suitable for testing most extreme obligate xerophiles, such as *A. halophilicus*, which would require even lower a_w_. Although glycerol can be used as the C source with many species, we obtained similar positive reactions in the tests performed in terms of changed/stained/cleared areas in at least some of the strains tested. While testing the use of cyclic or aliphatic hydrocarbons as C sources, solutes were required that could not be assimilated, so we used NaCl at a concentration with a similar a_w_.

### 4.3. Biodeterioration of Canvas and Proteins

According to previously published data on biodeteriorated paintings, the raw canvas on the *recto* side of the painting, which is potentially exposed to higher humidity behind paintings facing the wall [7], and/or the presence of proteins in the painting [22], are the critical factors for moulding in paintings. While the strong and diverse activities associated with cellulose degradation (endoglucanase, β-glucosidase, laccase) were abundant in xerotolerant *Cladosporium* species, obligately xerophilic *Aspergillus* and xerophilic *Penicillium* often expressed β-glucosidase but rarely endoglucanase. Surprisingly, the basidiomycetous taxa tested did not show strong cellulolytic activities. Fungi with laccase activity may also be involved in other biodegradation phenomena due to their non-specific activity, e.g., they may contribute to varnish and pigment degradation [74].

The genera *Aspergillus* and *Penicillium*, which are generally considered to be good producers of proteases potentially dangerous for proteinaceous paint layers adhesives [32], did not express or expressed strong gelatinase activity. Some *Cladosporium* strains were positive for caseinase. Fungal proteolytic activity is particularly dangerous for damaging natural binders and materials used in conservation procedures [32]. The results of different studies [15] were not always congruent in detected activities, which may indicate either strain specificity or that less strong activity was expressed in our study and not marked in the UpSet plot.

Linseed oil, the binder used in oil paintings, is a natural drying oil composed of a mixture of triglycerides derived primarily from unsaturated oleic, linoleic, and linolenic acids [10]. Esterases hydrolyse ester bonds in soluble and insoluble lipids in the binder of paintings and are the main contributors to the biodeterioration of paint layers [19,34]. Among the newly tested obligate xerophiles, the *A. salinicola* strain and one of the two *A. destruens* strains were positive for esterase activity, while all other obligate xerophiles were negative. Lipolytic and lecithinase activities were mainly attributed to *Aspergillus* and *Penicillium* strains. *Aspergillus proliferans*, *A. pseudoglaucus*, *P. bialowiezense*, *P. brevicompactum,* and *P. palitans* were indeed strong producers, with many species also expressing esterase activity.

### 4.4. Biodeterioration of Varnish and Wax

Traditionally, natural resins such as terpenoid resins, mastic, sandarac, copal, dammar, elemi, or rosin resins are used as varnishes for canvas paintings and are considered less acceptable for biodeterioration [34,35,36]. Various mixtures can also be used, such as terpenoid resin-based oil varnishes, which consist of a drying oil (linseed oil), a diterpenoid resin (colophony), and a triterpenoid resin (mastic) [34]. Due to their complex structure and composition, the degradation of varnishes requires combined enzymatic activities, such as esterase, urease, and possibly oxidising enzymes such as laccase and other lignin oxidases. The ability to grow on hydrocarbons (cyclic, aliphatic) may also contribute to varnish degradation. Several xerophilic fungi from our study had esterase and urease activity and the ability to use hydrocarbons as the sole carbon source: *P. chrysogenum*, *P. corylophilum*, *P. palitans*, *P. scabrosum*, *A. pullulans*, and *Parengyodontium album*. In fact, *P. chrysogenum* has already been reported to have these activities [75,76], and indeed, it can also degrade the sandarac (*Thuja articulata*) coating and induced changes in Manila Copal, both of which have been studied in mock-up experiments [35]. There have also been reports on the ability of *P. corylophilum* to degrade hydrocarbons [77], the urease activity and ability of *A. pullulans* to degrade hydrocarbons [78,79], and the urease activity of *P. album* [80]. None of the obligate xerophilic species possessed these abilities; although, there is a report on *A. destruens* degrading pollutant hydrocarbons under saline conditions [81].

Wax (beeswax), traditionally used in conservation–restoration to consolidate layers of paint, to line canvas, or as an additive to some varnishes, is a complex mixture of hydrocarbons, esters, and free fatty acids [82]. Fungi with high esterase activity and a strong ability to degrade hydrocarbons may also affect wax. In our study, these were numerous xerophilic *Penicillium* species (*P. bialowiezense*, *P. brevicompactum*, *P. chrysogenum*, *P. corylophilum*, *P. palitans,* and *P. scabrosum*), *A. conicus*, *A. reticulatus, A. jensenii*, *A. puulaauensis*, as well as xerotolerant *Coniochaeta ligninaria,* and *Parengyodontium album.* A painting with a protective layer of beeswax was deteriorated by an unidentified *Penicillium* species [83] and a wax seal by *Aureobasidium pullulans* and *Aspergillus*, *Penicillium*, and *Chaetomium* species [84,85]. Few *Penicillium* and *Cladosporium* species have also been isolated from wax drops on ancient manuscripts [86]. Recently, the growth of the xerophilic species *A. destruens*, *A. jensenii*, and the obligately xerophilic *Wallemia* species on canvas has been associated with wax [22], which could imply the involvement of different mechanisms of its degradation or co-metabolism, since only *A. jensenii* expressed all three activities listed, whereas *A. destruens* expressed only esterase and *Wallemia* only urease. However, as obligate xerophiles as single species do not possess the enzymatic activities mentioned, they are not considered to be the main degraders of these compounds.

### 4.5. Biodeterioration of Synthetic Polymers

Synthetic polymers, mainly polyesters, polyacrylics, and alkyd polymers, are the most stable materials used in cultural heritage art. However, in recent years it has become increasingly apparent that polymers can be susceptible to biodeterioration and incompatible with other materials [87,88,89,90]. Synthetic polymers can be used in a variety of ways: as canvas, as consolidants for paints, and as varnishes. Although there are some reports of fungal growth on these materials, the exact mechanisms of degradation are not yet known. Growth of certain species of the genera *Aspergillus*, *Aureobasidium*, *Cladosporium*, *Gliocladium,* and *Penicillium* has been observed on alkyd resins used as binders for pigments [8,91]. Members of all of these genera, as well as members of the genera *Aureobasidium*, *Cenococcum*, *Eladia*, *Epicoccum*, *Glyphium*, *Penicillium*, *Phoma,* and *Talaromyces,* have been identified on acrylic resins used for the restoration of various works of art [5,8,91,92,93,94,95]. The enzymes involved in degradation are esterases/lipases, proteases, and ureases with the ability to cleave ester bonds [96], together with the ability to degrade hydrocarbons, particularly polyurethane [97,98]. As fungi of the genus *Cladosporium* have been reported to grow well on alkyd and acrylic resins, a high level of esterase and urease activity, as well as the ability to degrade hydrocarbons, has been suggested [91,92,99]. This was confirmed in our study as all *Cladosporium* species showed strong esterase and urease activity and the ability to assimilate hexadecane. Similarly, *Chaetomium globosum*, previously reported to be able to grow on alkyd and acrylic resins [8,100], also showed these activities. *Aureobasidium pullulans*, which can grow on alkyd resin and polyurethane [91,95,101], showed strong esterase and urease activity in our study, in agreement with previous studies [101]. In conclusion, most xerophilic species of *Aspergillus* and *Penicillium* and xerotolerant species of *Aureobasidium* and *Cladosporium* in in our study showed esterase and urease activities, as well as the ability to assimilate hydrocarbons. Indeed, many species of *Aspergillus*, *Cladosporium,* and *Penicillium* are known degraders of plastics and have shown potential for use in bioremediation as good degraders of polycyclic aromatic hydrocarbons (PAHs) [77,81,102,103,104,105,106]. *Penicillium citrinum* is also commonly found on polyurethane and acrylic synthetic resins [95]. The xerophilic species *A. conicus* and possibly *A. infrequens* are also potential degraders of synthetic polymers.

### 4.6. Novel Tests for Biodeterioration Studies: Degradation of Environmental Debris and Ability to Sequester Fe Ions

Some oligotrophic fungi can live on the surfaces of paintings on dust deposits and various organic environmental debris, such as the remains of bacteria, spiders and their webs, and small arthropods, which can also act as microbial vectors [107]. DNA from dead organisms (bacteria, fungi, insects) is also a source of nucleotides, carbon, nitrogen, and phosphorus, made possible by the production of extracellular DNases. DNase activity is most often associated with fungal pathogenicity to animals and only in a few cases to saprobic taxa (reviewed in [108]). In our study, extracellular DNase activity was exhibited by all tested species of the genera *Aureobasidium*, *Cladosporium*, *Parengyodontium,* and most *Penicillium* and *Aspergillus* species, including the majority of extremely xerophilic species.

Various pigments can be sources of certain essential metals, such as iron, which can be biosequestered by synthesised microbial siderophores—ligands that chelate iron. This ability has been reported mainly in the context of microbial pathogenicity and is considered as an important virulence factor [45]. It was rarely detected in our study. Among the xerotolerant taxa, it was detected in the entomopathogenic *Akanthomyces muscarius* and *Beauveria pseudobassiana*, which could be in line with their pathogenicity, while the presence of siderophores in *A. conicus*, *A. destruens*, and in *A. domesticus* cannot be commented yet and requires further investigation.

## 5. Conclusions

Moderately xerophilic fungi of the genera *Penicillium*, *Aspergillus,* and *Cladosporium* show a high degree of metabolic versatility, possessing enzymes that allow the biodegradation of canvas and various paint layers, as well as environmental DNA. The greatest risk is posed by various *Penicillium* species such as *P. chrysogenum*, *P. scabrosum*, *P. palitans*, *P. brevicompactum*, and *P. bialowiezense*, which also excrete acids and pigments. Obligately xerophilic species, tested on media supplemented with glycerol to lower their a_w_, showed less diverse enzymatic activities. Nevertheless, they have the ability to grow under low RH conditions, where they can degrade the canvas and potentially affect the varnish and synthetic polymers, especially in combination with other taxa expressing lipase-esterase activities. Most of these species expressed DNase activity, indicating the importance of dust deposition for their establishment on painting surfaces. *Aspergillus domesticus* and *A. destruens* may affect paint layers and related synthetic materials. Interestingly, *A. vitricola* and *A. destruens*, which are the most common obligate xerophilic species on easel paintings [22], were found to degrade only a limited number of painting compounds, showing not only β-glucosidase and DNase activity but also excreting acids, so their biodeterioration potential should not be neglected and should be further investigated.

With only one exception, all of the isolates selected from the deteriorated paintings were xerotolerant or xerophilic and could grow at ambient temperatures (15–25 °C). Thirty-nine per cent of the strains also grew at 37 °C, indicating the need for precautionary measures for conservators–restorers when dealing with contaminated paintings.

Xerotolerant and moderately xerophilic fungi possess a wide range of enzymes, including urease, β-glucosidase, esterase, lecithinase, and DNase, which can potentially degrade all painting components under conditions of high water availability. The xerophilic strains that pose the greatest threat to paintings are *P. scabrosum*, *P. palitans*, *P. brevicompactum*, *P. bialowiezense,* and *P. chrysogenum*.

Obligate xerophiles had a narrower range of enzymatic activity, with β-glucosidase, DNase, urease, and endoglucanase being the most common enzymes.

Xerophilic fungi pose a threat to canvas paintings at low RH, especially to a canvas or partially degraded canvas, due to endoglucanase and β-glucosidase activity. Almost all strains tested can degrade environmental DNA through DNase activity. The greatest threat to paintings is posed by *Aspergillus penicillioides*, *A. salinicola*, *A. infrequens*, *Wallemia* aff. *Muriae*, *A. tardicrescens,* and *A. magnivesiculatus.*

Given the growth of obligate xerophilic fungi and their minimal growth requirements in terms of environmental conditions, this study provides additional evidence to the studies of Bastholm et al. (2022) [38] and Paiva de Carvalho et al. (2018) [69] that new measures should be considered in museums and painting repositories.

## Figures and Tables

**Table 1:** Performed enzyme tests with the main substrates, their producers, incubation conditions, and positive reactions of enzymatic assays.

		Incubation Conditions			
Enzymatic Activity	Substrate (Producer)	Temperature [°C]	Time [days]	Positive Reaction	Reference
amylase	soluble starch (Sigma-Aldrich)	24	14	a clear zone around colonies in black/blue stained medium after flooding with iodine solution	[49]
β-glucosidase	aesculin (6,7-dihydroxycoumarin 6-glucoside) (Merck)	24	14	a black zone around colonies in light-yellow medium	[49]
endocellulase	carboxymethyl cellulose (Sigma-Aldrich)	24	21	a yellow clear zone around colonies in red stained medium after flooding with Congo red and rinsing with NaCl solution	[49]
gelatinase	gelatine (Sigma-Aldrich)	24	14	liquefied medium around the colonies in yellow transparent medium	[49]
caseinase	skim milk (Merck)	24	14	a clear zone around colonies in opaque medium	[50]
esterase	Tween-80 (Sigma-Aldrich)	24	14	a purple/blue zone around colonies in yellow medium due to colour change in the indicator; precipitation of calcium salts	[51]
lecithinase	egg yolk	24	28	a white opaque zone around the colonies in yellow medium	[52]
lipase	tributyrin (Sigma-Aldrich)	24	28	a clear zone around the colonies in opaque medium	[53]
urease	urea (Merck)	24	14	change in colour of the medium from yellow to purple	[54]
laccase	ABTS (Sigma-Aldrich)	24	14	a purple halo around the colonies in light yellow transparent medium	[55]
laccase	guaiacol (Sigma-Aldrich)	24	14	an orange/brown halo around the colonies in light yellow transparent medium	[55]
DNase	DNase agar (Beckton and Dickinson)	24	7	a transparent halo around colonies in opaque white medium after flooding with 1 M HCl	[56]

## Data Availability

Data are contained within the article and supplementary materials.

## Supplementary Materials

The following supporting information can be downloaded at: https://www.mdpi.com/article/10.3390/jof10010076/s1, Table S1: Cultures used in this study, their source and identification. Table S2: Adaptation to low aw (xero-tolerance/-phily, halo-tolerance/-phily), growth at different temperatures, production of acids, and pigments of tested fungal species. Table S3: Enzymatic (amylase, endocellulase, β-glucosidase, caseinase, gelatinase, esterase, lipase, lecithinase, laccase, urease, and DNase), and other activities (assimilation of hydrocarbons, dissolution of CaCO_3_, and siderophore formation) of tested fungal species. Figure S1: Biodegradation assays with negative (upper row) and positive (lower row) enzymatic activities.
