# Supplementary material for: Degradation Potential of Xerophilic and Xerotolerant Fungi Contaminating Historic Canvas Paintings
Source: J Fungi (Basel). 2024 Jan 18;10(1):76. doi: 10.3390/jof10010076 (PMC10817455; doi:10.3390/jof10010076)
Supplement: Supplementary file 1 [file jof-10-00076-s001.zip › jof-2746199-supplementary.pdf]

Supplementary Material

Degradation potential of xerophilic and xerotolerant fungi contaminating historic canvas paintings

Amela Kujović, Cene Gostinčar, Katja Kavkler, Natalija Govedić, Nina Gunde-Cimerman, Polona Zalar* *** Correspondence:** Polona Zalar: polona.zalar@bf.uni-lj.si

1 Supplementary Tables

Table S1: Cultures used in this study, their source and identification.

EXF strain nr.	Fungal name	Source	Support	Paint	Isolation side	Identification DNA barcode: GenBank No.	Reference
10648	*Akanthomyces muscarius*	painting GM 1	flax canvas	acrylate	V	ITS: MW288713	Zalar et al. 2023
10559	*Alternaria* sp.	painting GBJ 2	unknown canvas	oil	V	ITS: MW288710	Zalar et al. 2023
10651	*Alternaria* sp.	painting GM 2	veneer	oil	V	ITS: MW288712	Zalar et al. 2023
7660	*Aspergillus conicus*	painting RCS 16	flax canvas	oil or greasy tempera	R	*BenA*: MW357091	Zalar et al. 2023
7677	*Aspergillus creber*	painting RCS 19	flax canvas	oil	R	*BenA*: MW357095	Zalar et al. 2023
10360	*Aspergillus destruens*	painting RCS 25	flax canvas	oil	V	*BenA*: MW369690	Zalar et al. 2023
7651	*Aspergillus destruens*	painting RCS 15	flax canvas	oil	R	*BenA*: MW369703	Zalar et al. 2023
10462	*Aspergillus domesticus*	painting RCS 24	flax canvas	oil	V	*BenA*: MW357175	Zalar et al. 2023
10450	*Aspergillus infrequens*	painting RCS 25	flax canvas	oil	V	*BenA*: MW357179	Zalar et al. 2023

EXF strain nr.	Fungal name	Source	Support	Paint	Isolation side	Identification DNA barcode: GenBank No.	Reference
10405	*Aspergillus jensenii*	painting RCS 15	flax canvas	oil	R	*BenA*: MW357112	Zalar et al. 2023
10353	*Aspergillus magnivesiculatus*	painting RCS 24	flax canvas	oil	V	*BenA*: MW357145	Zalar et al. 2023
10425	*Aspergillus penicillioides*	painting RCS 24	flax canvas	oil	V	*BenA*: MW357162	Zalar et al. 2023
15555	*Aspergillus proliferans*	painting ART-4	unknown canvas	oil	R	*BenA*: PP034129	Zalar et al. 2023
15119	*Aspergillus pseudoglaucus*	painting RCS 22	hemp and flax canvas	oil or greasy tempera	V	*BenA*: MW357166	Zalar et al. 2023
14316	*Aspergillus pseudoglaucus*	mock up sample contamination	wood impregnated with animal glue, oil paint, and Regalrez 1126	oil	V	*BenA*: PP034128	this study
7678	*Aspergillus puulaauensis*	painting RCS 19	flax canvas	oil	R	*BenA*: MW357120	Zalar et al. 2023
10429	*Aspergillus reticulatus*	painting RCS 21	flax canvas	oil or greasy tempera	R	*BenA*: MW357169	Zalar et al. 2023
10401	*Aspergillus salinicola*	painting RCS 22	hemp and flax canvas	oil or greasy tempera	V	*BenA*: MW357181	Zalar et al. 2023
7656	*Aspergillus sloanii*	painting RCS 16	flax canvas	oil or greasy tempera	R	*BenA*: MW357182	Zalar et al. 2023
10431	*Aspergillus tardicrescens*	painting RCS 25	flax canvas	oil	R	*BenA*: MW357143	Zalar et al. 2023
10463	*Aspergillus vitricola*	painting RCS 26	flax canvas	oil	R	*BenA*: MW357199	Zalar et al. 2023
15210	*Aspergillus vitricola*	painting RCS 22	hemp canvas	oil or greasy tempera	R	ITS: PP033020	Zalar et al. 2023
10372	*Aureobasidium melanogenum*	air in RCS depository	-	-	-	ITS: MW288717	Zalar et al. 2023
14317	*Aureobasidium pullulans*	mock up sample contamination	wood, animal glue, oil paint, Beva 371	oil	V	ITS: PP033021	this study
10556	*Beauveria pseudobassiana*	painting GBJ 2	unknown canvas	oil	V	ITS: MW288719	Zalar et al. 2023
15111	*Bjerkandera adusta*	painting RCS 22	hemp and flax canvas	oil or greasy tempera	V	ITS: MW288722	Zalar et al. 2023
10840	*Botryotrichum domesticum*	painting RCS 24	flax canvas	oil	V	ITS: MW288937	Zalar et al. 2023
7669	*Chaetomium cochlioides*	painting RCS 16	flax canvas	oil or greasy tempera	R	ITS: MW288932	Zalar et al. 2023
7690	*Chaetomium globosum*	painting RCS 18	hemp canvas	oil	R	ITS: MW288933	Zalar et al. 2023
10573	*Cladosporium allicinum*	painting GBJ 1	plywood	oil	V	*act*: MW387133	Zalar et al. 2023
14315	*Cladosporium cladosporioides*	mock up sample contamination	glass impregnated with Regalrez 1126	-	-	*act*: PP034131	this study

EXF strain nr.	Fungal name	Source	Support	Paint	Isolation side	Identification DNA barcode: GenBank No.	Reference
10496	*Cladosporium pseudocladosporioides*	painting GBJ 2	unknown canvas	oil	V	*act*: MW369723	Zalar et al. 2023
14314	*Cladosporium pseudocladosporioides*	mock up sample contamination	glass impregnated with Paraloid B72	-	V	*act*: PP034132	this study
10492	*Cladosporium westerdijkiae*	painting GBJ 1	plywood	oil	V	*tef1*: MW387144	Zalar et al. 2023
10656	*Cladosporium xylophilum*	painting GM 2	veneer	oil	V	*tef1*: MW387151	Zalar et al. 2023
15505	*Coniochaeta ligninaria*	painting ART-4	unknown canvas	oil	V	ITS: OR097691	Zalar et al. 2023
15086	*Cylindrobasidium sp.*	painting ART-1	flax canvas	oil	V	ITS: MW288728	Zalar et al. 2023
10652	*Debaryomyces hansenii*	painting GM 2	veneer	oil	V	ITS: MW288763	Zalar et al. 2023
10694	*Parengyodontium album*	Celje ceiling	linen canvas	tempera	V	ITS: MZ687377	Kavkler et al. 2022
10663	*Parengyodontium album*	Celje ceiling	linen canvas	tempera	V	ITS: MZ687373	Kavkler et al. 2022
10693	*Parengyodontium album*	Celje ceiling	linen canvas	tempera	V	ITS: MZ687376	Kavkler et al. 2022
10584	*Penicillium bialowiezense*	painting RCS 15	flax canvas	oil	V	*BenA*: MW357228	Zalar et al. 2023
10484	*Penicillium brevicompactum*	painting GBJ 1	plywood	oil	V	*BenA*: MW357229	Zalar et al. 2023
10495	*Penicillium chrysogenum*	painting GBJ 2	unknown canvas	oil	V	*BenA*: MW357233	Zalar et al. 2023
15508	*Penicillium chrysogenum*	painting ART-4	unknown canvas	oil	V	*BenA*: PP034130	Zalar et al. 2023
7655	*Penicillium corylophilum*	painting RCS 16	flax canvas	oil or greasy tempera	V	*BenA*: MW357217	Zalar et al. 2023
15064	*Penicillium corylophilum*	painting ART-1	flax canvas	oil	V	*BenA*: MW357214	Zalar et al. 2023
10368	*Penicillium palitans*	painting RCS 26	flax canvas	oil	V	*BenA*: MW357080	Zalar et al. 2023
10503	*Penicillium rubens*	painting GBJ 2	unknown canvas	oil	V	*BenA*: MW357086	Zalar et al. 2023
10557	*Penicillium scabrosum*	painting GBJ 2	unknown canvas	oil	V	*BenA*: MW357087	Zalar et al. 2023
15049	*Trametes versicolor*	air, RCS depository	-	-	-	ITS: MW288749	Zalar et al. 2023
10502	*Trichoderma longibrachiatum*	painting GBJ 2	unknown canvas	oil	V	ITS: MW288751	Zalar et al. 2023
10120	*Wallemia* aff. *muriae*	painting RCS 20	flax canvas	oil and greasy tempera	V	ITS: MW288065	Zalar et al. 2023
10342	*Wallemia canadensis*	painting RCS 21	flax canvas	oil and greasy tempera	R	ITS: MW288071	Zalar et al. 2023
10650	*Zalaria obscura*	painting GM 2	veneer	oil	V	ITS: MW288752	Zalar et al. 2023

Legend: GM: Regional Museum Goriški muzej, Solkan (Slovenia); GB: Božidar Jakac Art Museum, Kostanjevica na Krki (Slovenia); RCS: Restoration Centre, Institute for the Protection of Cultural Heritage of Slovenia, Ljubljana (Slovenia); ART-1: painting in church, Koper (Slovenia); ART-4: painting in church, Leskovec at Višnja gora (Slovenia). V: isolated from the verso (front side) of the painting; R: isolated from the recto (back side) of the painting. ITS, internal transcribed spacer region including intervening 5.8S rRNA gene; *act*, partial actin gene; *tef1*, partial translation elongation factor 1-alpha gene; *BenA*, partial beta-tubulin gene; For more information on paintings we refer to Zalar et al. (2023). EXF - designation of fungal strains in Ex, Microbial Culture Collection of the Department of Biology, Biotechnical Faculty, University of Ljubljana (Infrastructural Centre Mycosmo, MRIC UL, Slovenia).

References:

Zalar, P.; Graf Hriberšek, D.; Gostinčar, C.; Breskvar, M.; Džeroski, S.; Matul, M.; Novak Babič, M.; Čremožnik Zupančič, J.; Kujović, A.; Gunde-Cimerman, N.; et al. Xerophilic Fungi Contaminating Historically Valuable Easel Paintings from Slovenia. Front. Microbiol. 2023, 14, doi:10.3389/fmicb.2023.1258670.

Kavkler, K.; Humar, M.; Kržišnik, D.; Turk, M.; Tavzes, Č.; Gostinčar, C.; Džeroski, S.; Popov, S.; Penko, A.; Gunde - Cimerman, N.; et al. A Multidisciplinary Study of Biodeteriorated Celje Ceiling, a Tempera Painting on Canvas. Int. Biodeterior. Biodegradation 2022, 170, 105389, doi:https://doi.org/10.1016/j.ibiod.2022.105389.

Table S2: Adaptation to low a_w (xero-tolerance/-phily, halo-tolerance/-phily), growth at different temperatures, production of acids and pigments of tested fungal species.

EXF	Fungal name	XERO	HALO	TEMP	pH	PIGM
10648	*Akanthomyces muscarius*	1	2	2	0	0
10559	*Alternaria* sp.	2	1	2	0	1
10651	*Alternaria* sp.	2	2	2	0	0
7660	*Aspergillus conicus*	3	3	2	0^	0
7677	*Aspergillus creber*	3	3	2	0^	0
10360	*Aspergillus destruens*	**3**	3	2	0^	0
**7651**	***Aspergillus destruens***	**4**	4	2	0^	0
**10462**	***Aspergillus domesticus***	4	4	1	0^	0
**10450**	***Aspergillus infrequens***	4	4	3	0^	0
10405	*Aspergillus jensenii*	3	3	2	0^	1
**10353**	***Aspergillus magnivesiculatus***	4	4	4	0^	0
**10425**	***Aspergillus penicillioides***	4	4	4	0^	0
15555	*Aspergillus proliferans*	3	5	1	0	1
15119	*Aspergillus pseudoglaucus*	3	3	2	0^	1
14316	*Aspergillus pseudoglaucus*	3	3	2	0	1
7678	*Aspergillus puulaauensis*	3	3	2	0^	0
10429	*Aspergillus reticulatus*	3	3	2	0^	0
**10401**	***Aspergillus salinicola***	5	3	2	0^	0
**7656**	***Aspergillus sloanii***	4	3	2	0^	0
**10431**	***Aspergillus tardicrescens***	4	4	2	0^	0
**10463**	***Aspergillus vitricola***	4	4	2	0^	0
**15210**	***Aspergillus vitricola***	4	4	3	0^	0
10372	*Aureobasidium melanogenum*	3	2	2	0	0
14317	*Aureobasidium pullulans*	2	3	2	0	0
10556	*Beauveria pseudobassiana*	1	1	2	0	0
15111	*Bjerkandera adusta*	0	0	3	0	0
10840	*Botryotrichum domesticum*	1	0	2	0	0
7669	*Chaetomium cochlioides*	1	1	2	0	0
7690	*Chaetomium globosum*	1	0	2	0	1
10573	*Cladosporium allicinum*	2	2	3	0	0
14315	*Cladosporium cladosporioides*	3	2	2	0	1
10496	*Cladosporium pseudocladosporioides*	2	2	2	0	0
14314	*Cladosporium pseudocladosporioides*	3	2	3	0	0
10492	*Cladosporium westerdijkiae*	2	2	3	0	0
10656	*Cladosporium xylophilum*	2	2	3	0	0
15505	*Coniochaeta ligniaria*	1	1	3	0	0
15086	*Cylindrobasidium* sp.	1	0	3	0	0
10652	*Debaryomyces hansenii*	3	3	3	0	0
10694	*Parengyodontium album*	1	2	3	0	0

10663	*Parengyodontium album*	1	2	2	0	0
10693	*Parengyodontium album*	1	2	3	0	0
10584	*Penicillium bialowiezense*	3	3	2	1	0
10484	*Penicillium brevicompactum*	3	3	2	1	0
10495	*Penicillium chrysogenum*	3	3	3	2	1
15508	*Penicillium chrysogenum*	3	3	3	2	1
7655	*Penicillium corylophilum*	3	3	2	1	0
15064	*Penicillium corylophilum*	3	3	3	1	1
10368	*Penicillium palitans*	3	3	2	2	0
10503	*Penicillium rubens*	3	3	3	1	1
10557	*Penicillium scabrosum*	3	3	2	2	1
15049	*Trametes versicolor*	1	0	3	0	0
10502	*Trichoderma longibrachiatum*	1	1	3	0	1
**10120**	***Wallemia* aff. *muriae***	4	4	2	0^	0
10342	*Wallemia canadensis*	3	3	2	0^	0
10650	*Zalaria obscura*	2	3	3	0	0

Legend: Activities are shown as groups according to four criteria: (i) 0-5 shown in red, (ii) 1-4 shown in green, (iii) 0-2 shown in blue, (iv) 0-1 shown in yellow; 0 = no activity; 1, 2 or 5 = maximum activities, except for the green criteria; XERO: xerophily; 0= non-xerotolerant fungi (growth only in medium without added glycerol); 1= weakly xerotolerant (growth on agar media without and with 18% glycerol); 2= moderately xerotolerant; 3= moderately xerophilic (growth on all tested agar media); 4= obligately or extremely xerophilic fungi growing on 18% glycerol; 5= obligately or extremely xerophilic fungi growing only on 30 and 40% glycerol. HALO: halophily; 0= non-halotolerant (no growth on media supplemented with 10% NaCl); 1= weakly halotolerant (growth without and with 10% NaCl); 2= moderately halotolerant (growth without and with maximally 15% NaCl); 3= moderately halophilic (growth at 10, 20, and 25% NaCl); 4= obligately or extremely halophilic fungi growing only in the presence of 10 or 15% NaCl; and 5= obligately or extremely halophilic fungi growing at minimally 15% NaCl. TEMP: temperature test (1=growth at 15 °C, no growth at 24 and 37 °C; 2= growth at 15 °C and 24 °C, no growth at 37 °C; 3= growth at 15, 24 and 37 °C; 4= growth at 24 and 37 °C, no growth at 15 °C. pH: acid production (0= no change in pH, final pH 6.0–6.1; 1= final pH 5.0–5.9; 2= final pH 4.0–4.9). PIGM: production of soluble pigments (0= no production; 1= pigment production), ^ addition of 10% NaCl. Strain numbers in bold: obligate or extreme xerophiles.

Table S3: Enzymatic (amylase, endocellulase, β-glucosidase, caseinase, gelatinase, esterase, lipase, lecithinase, laccase, urease, and DNase) and other activities (assimilation of hydrocarbons, dissolution of $CaCO_3$ and siderophore formation) of tested fungal species.

EXF	Fungal name	AMY	ENDO	β-GLU	CAS	GEL	ES	LIP	LEC	LAC gua	LAC abts	URE	DNA	$CaCO_3$	HEX	MO	SID
10648	*Akanthomyces muscarius*	1	0	5	1	0	1	0	0	0	0	1	1	2	2	2	4
10559	*Alternaria* sp.	1	1	2	1	1	1	0	1	0	0	0	0	0	2	0	0^
10651	*Alternaria* sp.	1	1	1	1	0	1	0	1	0	0	1	2	0	2	0	0
7660	*Aspergillus conicus*	0*	4*	5*	0*	0*	2	0	0	0*	0*	1	0*	1*	2°	0°	3*^
7677	*Aspergillus creber*	1*	1	1*	1*	2*	1	3	1	0*	0*	1*	3	0	0°	1°	0
7651	*Aspergillus destruens*	1*	0*	4*	0*	0*	0	/	0	0*	0*	0*	1*^	1*	0°	0°	0*
**10360**	***Aspergillus destruens***	0*	2*	4*	0*	0*	5	0	0	0*	0*	0*	0*	1*	0°	0°	5*^
**10462**	***Aspergillus domesticus***	1*	1*	3*	0*	0*	0*	0	0	0*	0*	0*	4*^	1*	1°	0°	5*^
**10450**	***Aspergillus infrequens***	1*	0	3*	0*	0*	0	/	0	0*	0*	1*	3*^	1	2°	0°	0*
10405	*Aspergillus jensenii*	1*	5*	1*	0*	5	4	2	1	0*	0*	1*	4	0	0°	2°	0^
**10353**	***Aspergillus magnivesiculatus***	1*	2*	1*	0*	0*	0*	/	0	0*	0*	1*	3*	0*	1°	0°	0*
**10425**	***Aspergillus penicillioides***	/	3*	3*	0*	0*	0*	3	1	0*	0*	1*	3*^	0*	0°	0°	0*
15555	*Aspergillus proliferans*	0	0	1*	1*	1*	0	5	1	0*	0*	1	0*	0	0°	0°	0*
15119	*Aspergillus pseudoglaucus*	1*	0	1*	0*	0*	3	2	1	0	0	0*	2	1	0°	0°	0
14316	*Aspergillus pseudoglaucus*	0	0	4*	0*	0*	4	2	1	0	0	0*	1	1	0°	0°	0
7678	*Aspergillus puulaauensis*	2*	2	1*	0*	0	3	1	1	0	0	1*	3	0	0°	2°	0
10429	*Aspergillus reticulatus*	1*	2*	3*	0*	0*	5	0	0	0*	0*	1*	0*	1*	2°	0°	0*
**10401**	***Aspergillus salinicola***	1*	0	1*	0*	0*	3	2	1	0*	0*	1*	3*	0*	0°	0°	0*
**7656**	***Aspergillus sloanii***	1	1*	2	0*	0*	0*	0	1	0*	0*	1*	0*	0	0°	0°	0*
**10431**	***Aspergillus tardicrescens***	1*	4*	3*	0*	0*	0*	/	0	0*	0*	0*	3*^	1*	0°	0°	0*
**10463**	***Aspergillus vitricola***	0*	0*	1*	0*	0*	0*	/	0	0*	0*	0*	5*^	1*	0°	0°	0*
**15210**	***Aspergillus vitricola***	0*	1*	2*	0*	0*	0*	/	0	0*	0*	0*	0*	0	0°	0°	0*
10372	*Aureobasidium melanogenum*	1	2	2	4	0	5	1	0	0	0	1	4	2	1	0	0
14317	*Aureobasidium pullulans*	4	2	2	0	2	5	4	1	0	0	1	5	2	0	0	0
10556	*Beauveria pseudobassiana*	1	0	4	0	0	1	0	0	3	5	1	0	1	2	2	1
15111	*Bjerkandera adusta*	1	0	0	1	1	0	0	0	0	0	0	0	1	0	0	0
10840	*Botryotrichum domesticum*	2	1	2	0	0	1	0	0	0	0	1	2	0	2	0	0
7669	*Chaetomium cochlioides*	0	1	1	0	/	1	0	0	0	0	1	0	0	2^	1^	0
7690	*Chaetomium globosum*	1	1	2	0	/	1	0	0	0	0	1	0	0	2	1	0
10573	*Cladosporium allicinum*	3	2	4	3	1	5	0	0	3	2	1	4	0	1	0	0
14315	*Cladosporium cladosporioides*	1	2	2	2	1	3	1	1	5	5	1	4	0	1	0	0
10496	*Cladosporium pseudocladosporioides*	2	2	2	1	0	5	0	0	0	1	1	2	0	1	0	0

14314	*Cladosporium pseudocladosporioides*	3	2	2	4	0	5	/	0	0	0	1	3	0	1	0	0
10492	*Cladosporium westerdijkiae*	1	2	3	1	1	3	0	0	2	3	1	1	0	1	0	0
10656	*Cladosporium xylophilum*	1	2	4	1	0	5	0	0	0	0	1	2	0	1	0	0
15505	*Coniochaeta ligninaria*	1	1	2	1	0	3	0	1	0	0	1	4^	1	2^	0^	0
15086	*Cylindrobasidium* sp.	/	0	1	1	2	1	0	0	0	0	0	0	0	0	0	0
10652	*Debaryomyces hansenii*	0	0	5	0	0	5	0	0	0	0	1^	0	0	0	0	0
10663	*Parengyodontium album*	1	1	3	5	0	3	2	1	0	0	1	2	0	2^	2^	0
10693	*Parengyodontium album*	1	1	3	1	0	3	3	1	0	0	1	2	0	2^	2^	0
10694	*Parengyodontium album*	1	1	4	1	0	1	1	1	0	0	1	3	0	2^	2^	0
10584	*Penicillium bialowiezense*	1*	5*	4	0*	2	3	2	1	0	0	1	5	2	2	2	0
10484	*Penicillium brevicompactum*	4	3	4	5	1	4	2	1	0	0	1	5	1	2	1	5
10495	*Penicillium chrysogenum*	5	1	2	0	2	1	1	1	0	0	1	2	2	2	2	1
15508	*Penicillium chrysogenum*	4	1	2	0	2	2	0	1	0	0	1	3	2	2	0	2
7655	*Penicillium corylophilum*	2	0	3	1	0	4	0	0	0	0	0^	1	0	2	2	2^
15064	*Penicillium corylophilum*	1	0	5	1^	0	2	0	1	0	0	1	1	0	2	2	0
10368	*Penicillium palitans*	5	2	4	0	0	2	2	1	0	0	1	1	2	2	2	4
10503	*Penicillium rubens*	4	1	1	0	4	1	0	1	0	0	1	1	2	2	2	1
10557	*Penicillium scabrosum*	2	1	3	5	5	2	1	1	0	0	1	4	1	2	2	2
15049	*Trametes versicolor*	0	0	1	0	0	0	0	0	0	1	0	0	0	1	0	0
10502	*Trichoderma longibrachiatum*	1	1	1	1	2	1	0	1	0	0	1	2	0	0	0	0^
**10120**	***Wallemia* aff. *muriae***	0*	5*	3*	0*	0*	0*	/	0	0*	0*	1*	0*	0	0°	0°	0*
10342	*Wallemia canadensis*	0*	5*	4	0*	0*	2	0	0	0*	/	1*	0*	0	0°	0°	0*
10650	*Zalaria obscura*	5	0	5	0	0	2	0	0	0	0	1	0	2	0	0	0

Legend: Activities are shown as factors according to three criteria: (i) 0-5 shown in red, (ii) 0-1 shown in yellow and (iii) 0-2 shown in blue; 0 = no activity, maximum value (1,2 or 5) = maximum activity. AMY: amylase; ENDO: endocellulase; β-GLU: β-glucosidase; CAS: caseinase; GEL: gelatinase; ES: esterase, LIP: lipase; LEC: lecithinase; LAC: laccase (GUA: guaiacol, abts: ABTS); URE: urease; DNA: DNase; $CaCO_3$: carbonate ($CaCO_3$) dissolution; HEX: assimilation of hexadecane, MO: assimilation of mineral oil; SID: siderophores formation. * reduced water activity with addition of 18% glycerol; ° reduced water activity with addition of 10% NaCl; ^ prolonged incubation; / no information; extreme values are underlined. Obligately or extremely xerophilic species are bolded.

2 Supplementary Figures

Figure S1. Biodegradation assays with negative (upper row) and positive (lower row) enzymatic activities.

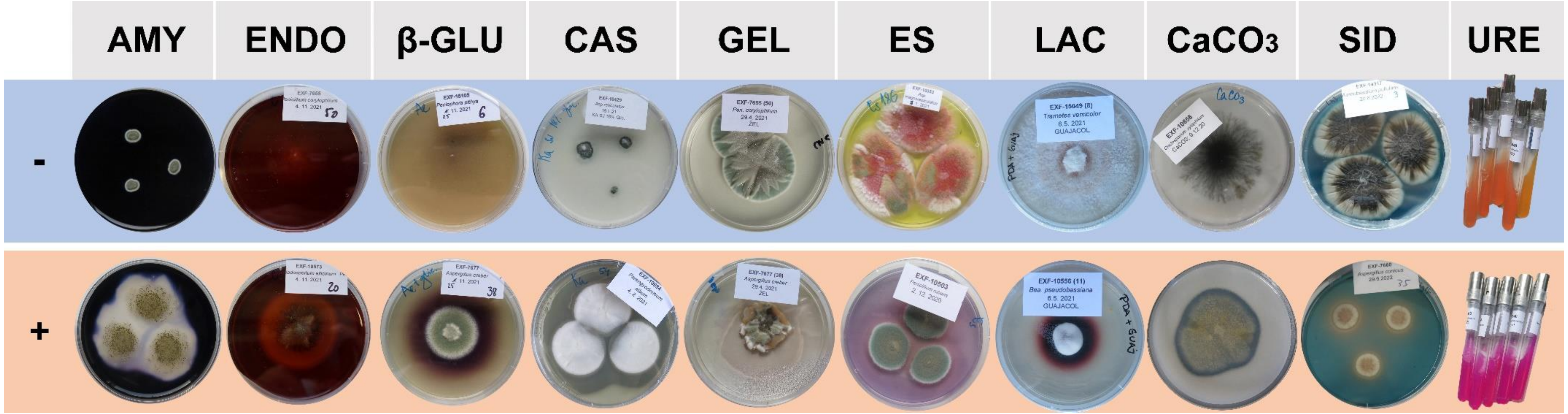

Legend: AMY: amylase - white unstained area around colonies in the otherwise violet medium dyed with iodine solution indicates starch degradation; ENDO: endoglucanase - light red halo on CMC agar dyed with Congo Red indicates cellulose degradation; β-GLU: β-glucosidase - black precipitate indicates β-glucosidase activity on aesculin agar; CAS: caseinase - transparent zone in otherwise opaque medium indicates casein degradation; GEL: gelatinase - solubilization of gelatine; ES: esterase - color change of pH indicator from yellow to purple indicates esterase activity; LAC: laccase - red halo indicates laccase activity on agar containing guaiacol; $CaCO_3$: $CaCO_3$ solubilization - transparent zone in otherwise opaque medium indicates carbonate dissolution; SID: siderophore production - orange stained areas around colonies indicate synthesis of siderophores; URE: urease - change of pH indicator from orange to purple indicates urea degradation.
